# Residential Levels of Polybrominated Diphenyl Ethers and Risk of Childhood Acute Lymphoblastic Leukemia in California

**DOI:** 10.1289/ehp.1307602

**Published:** 2014-06-03

**Authors:** Mary H. Ward, Joanne S. Colt, Nicole C. Deziel, Todd P. Whitehead, Peggy Reynolds, Robert B. Gunier, Marcia Nishioka, Gary V. Dahl, Stephen M. Rappaport, Patricia A. Buffler, Catherine Metayer

**Affiliations:** 1 Occupational and Environmental Epidemiology Branch, Division of Cancer Epidemiology and Genetics, National Cancer Institute, National Institutes of Health, Department of Health and Human Services, Bethesda, Maryland, USA; 2 University of California, Berkeley, School of Public Health, Berkeley, California, USA; 3 Cancer Prevention Institute of California, Berkeley, California, USA; 4 Battelle Memorial Institute, Columbus, Ohio, USA; 5 Lucile Packard Children’s Hospital at Stanford University, Palo Alto, California, USA; * These authors contributed equally to this work.

## Abstract

Background: House dust is a major source of exposure to polybrominated diphenyl ethers (PBDEs), which are found at high levels in U.S. homes.

Methods: We studied 167 acute lymphoblastic leukemia (ALL) cases 0–7 years of age and 214 birth certificate controls matched on date of birth, sex, and race/ethnicity from the Northern California Childhood Leukemia Study. In 2001–2007, we sampled carpets in the room where the child spent the most time while awake; we used a high-volume small-surface sampler or we took dust from the home vacuum. We measured concentrations of 14 PBDE congeners including penta (28, 47, 99, 100, 153, 154), octa (183, 196, 197, 203), and decaBDEs (206–209). Odds ratios (ORs) were calculated using logistic regression, adjusting for demographics, income, year of dust collection, and sampling method.

Results: BDE-47, BDE-99, and BDE-209 were found at the highest concentrations (medians, 1,173, 1,579, and 938 ng/g, respectively). Comparing the highest to lowest quartile, we found no association with ALL for summed pentaBDEs (OR = 0.7; 95% CI: 0.4, 1.3), octaBDEs (OR = 1.3; 95% CI: 0.7, 2.3), or decaBDEs (OR = 1.0; 95% CI: 0.6, 1.8). Comparing homes in the highest concentration (nanograms per gram) tertile to those with no detections, we observed significantly increased ALL risk for BDE-196 (OR = 2.1; 95% CI: 1.1, 3.8), BDE-203 (OR = 2.0; 95% CI: 1.1, 3.6), BDE-206 (OR = 2.1; 95% CI: 1.1, 3.9), and BDE-207 (OR = 2.0; 95% CI: 1.03, 3.8).

Conclusion: We found no association with ALL for common PBDEs, but we observed positive associations for specific octa and nonaBDEs. Additional studies with repeated sampling and biological measures would be informative.

Citation: Ward MH, Colt JS, Deziel NC, Whitehead TP, Reynolds P, Gunier RB, Nishioka M, Dahl GV, Rappaport SM, Buffler PA, Metayer C. 2014. Residential levels of polybrominated diphenyl ethers and risk of childhood acute lymphoblastic leukemia in California. Environ Health Perspect 122:1110–1116; http://dx.doi.org/10.1289/ehp.1307602

## Introduction

Acute lymphoblastic leukemia (ALL) is the most common form of childhood cancer, accounting for about 80% of childhood leukemia in most Western countries (Ross and Spector 2006). Incidence peaks at 2–5 years of age, and in the United States rates are highest among Hispanics (Ries et al. 2004). Childhood leukemia incidence is generally higher in industrialized countries than in developing ones (Ross and Spector 2006). The international variation and the early age peak in incidence suggest that environmental exposures during pregnancy or early life are important to disease development; however, to date, few risk factors for ALL have been identified (Buffler et al. 2005).

Polybrominated diphenyl ethers (PBDEs) are flame-retardant chemicals used in electronics, furniture, and some textiles (Birnbaum and Staskal 2004). They are structurally similar to polychlorinated biphenyls (PCBs), are persistent in the environment, and bioaccumulate through the food chain [U.S. Environmental Protection Agency (EPA) 2012]. Use of the three commercial formulations—pentaBDE, octaBDE, and decaBDE (named for the average number of bromines in the congeners of each formulation)—increased in the United States from the 1980s until 2004, when U.S. manufacturing of penta and octaBDE was voluntarily phased out due to concerns about toxicity (U.S. EPA 2008, 2012). DecaBDE was voluntarily phased out in the United States at the end of 2013. However, because of their persistence and their continued use in some imported products (U.S. EPA 2013), human exposure to PBDEs continues. Further, decaBDE can break down in the environment into more bioavailable PBDE congeners (Söderstrom et al. 2004; Stapleton and Dodder 2008), which may result in continued exposure to lower brominated PBDEs.

The primary sources of PBDEs are consumer products including plastic housings of computers and other electronics, foam upholstery, carpet pads, and mattresses (Birnbaum and Staskal 2004; U.S. EPA 2012). Because PBDEs are not chemically bound to treated products and often constitute several percent by weight, they can migrate into the indoor environment resulting in high indoor concentrations (Birnbaum and Staskal 2004). Approximately 80% of PBDE exposure in the United States is estimated to come from nonfood sources (Lorber 2008), and ingestion of and dermal contact with PBDEs in house dust is a major route of exposure (Coakley et al. 2013; Lorber 2008; Stapleton et al. 2012; Watkins et al. 2012; Wu et al. 2007). Young children tend to have higher PBDE body burdens than adults (Rose et al. 2010; Sjödin et al. 2008) most likely because of their smaller body size and greater intake of dust through frequent hand-to-mouth activity (Harrad et al. 2010; Lorber 2008; Tulve et al. 2002). Further, levels in California children are among the highest in the world because California has had the strictest fire retardant standards for consumer products in the country (Bradman et al. 2012; Rose et al. 2010).

Environmental chemical exposures that are hematotoxic, damage DNA, or interfere with immune system function are of interest as possible leukemia risk factors (Buffler et al. 2005; Ross and Spector 2006). A cross-sectional study of adolescents in the Netherlands found that higher serum concentrations of PBDEs were associated with decreased numbers of lymphocytes (Leijs et al. 2009). Animal studies have shown that PBDEs cause perturbations of cytokine and chemokine levels (Lundgren et al. 2009), increases in lymphocyte proliferation, and decreases in peripheral monocytes and natural killer (NK) cell activity (Fair et al. 2012). Moreover, exposure to PBDEs has been associated with adverse neurological outcomes in children (Eskenazi et al. 2013; Herbstman et al. 2010; Roze et al. 2009), congenital cryptorchidism (Main et al. 2010), and alteration of thyroid hormones in adults (Turyk et al. 2008).

Previously, we reported that PCBs in house dust were positively associated with childhood ALL in California (Ward et al. 2009). Because PBDEs are structurally similar to PCBs and are present at high concentrations in California homes, we also analyzed household dust samples for 14 PBDEs. To our knowledge, no prior epidemiologic studies have evaluated residential PBDEs and childhood leukemia risk.

## Methods

*Study population*. We conducted a population-based case–control study of childhood leukemia in Northern and Central California (the Northern California Childhood Leukemia Study [NCCLS]), which included 17 counties in the San Francisco Bay area and 18 counties in the Central Valley (1995–2008) (Bartley et al. 2010). Briefly, cases ≤ 14 years of age were identified from the nine major pediatric clinical centers in the study area. Controls were selected from California birth certificate files and were individually matched (initially 2:1 and then later 1:1 due to funding limitations) to cases on date of birth, sex, maternal race, Hispanic ethnicity, and maternal residence in the study area. Interviews of cases and controls in the main study (tier 1) included questions about child care attendance, residential history, parental occupation, smoking, and other factors. Cases and controls who were < 8 years old at diagnosis/reference date (December 1999–June 2006) and still living in the homes they occupied at the diagnosis/reference date were eligible for a second interview and dust sampling in the home (tier 2).

Eligibility was limited to younger cases and controls so that the dust sample reflected residential exposures over a substantial portion of the child’s life. Because of the requirement that cases and controls were living in the diagnosis/reference home, individual matching of cases and controls was not maintained; instead, analyses were adjusted for the matching factors.

The tier 2 interviews (2001–2007) included questions about household pesticide use, home age and type, carpet age, and parental occupations. A global positioning system measurement of the home location was taken, and we determined whether the residence was located in an urban, suburban, or rural area based on the 2000 U.S. census.

Carpet dust samples were collected primarily using a specialized vacuum, the high-volume small-surface sampler (HVS3; Envirometrics, Inc.), in the room where the child spent the most time while awake. We sampled one or more carpets/rugs that were at least 1 m^2^ in size and were present before the reference date (Colt et al. 2008; Ward et al. 2009). Most samples were from the living or family room. Interviewers also collected existing dust from the household vacuum cleaner. HVS3 dust was analyzed preferentially, with household vacuum dust used only when there was insufficient HVS3 dust. After we determined that HVS3 and household vacuum dust concentrations of pesticides, PCBs, and polycyclic aromatic hydrocarbons were highly correlated (Colt et al. 2008), we sampled dust only from household vacuums from July 2006 through 2007 in lieu of the more time-intensive HVS3 method.

During the tier 2 study period, the participation rate for the first interview among families of cases and controls < 8 years old was 86% (Metayer et al. 2013). A total of 324 cases and 407 controls met the eligibility criteria for tier 2; 296 leukemia cases (91%) including 269 ALLs, and 333 controls (82%) participated. Among those with approximately ≥ 0.5 g of dust, we selected all cases and controls with HVS3 dust and a sample of those with household vacuum dust, giving 167 ALL cases and 214 controls for PBDE analysis. HVS3 dust was analyzed for 101 (61%) cases and 133 (62%) controls. Because of the longer time needed to enroll birth certificate controls in the main study, the time between the reference date and dust collection was shorter for cases [median years, interquartile range (IQR): 0.92 (0.74–1.26] than for controls [1.66 (1.26–2.24)]. The study was reviewed by institutional review boards at the University of California, Berkeley, and the National Cancer Institute. Participants (child’s parent or legal guardian) gave written informed consent before the interviews and dust collection.

*Laboratory methods*. PBDEs were analyzed by the same laboratory that was used previously for PCBs, pesticides, and other chemicals; details of the sample processing have been described (Colt et al. 2008). Dust samples were sieved and the fine fraction (< 150 μm) retained. A 0.1-g portion of each dust sample was analyzed for 14 PBDEs in batches with 15 participants’ samples, one participant’s sample in duplicate, the same sample spiked with low and high concentrations of all analytes, and a solvent method blank. All batches included at least 4 case and 4 control samples; laboratory personnel were blind to case/control status. Each dust sample was spiked with 250 ng of the following Surrogate Recovery Standards (SRS) that are not found in commercial flame retardant mixtures or are at trace levels (LaGuardia et al. 2006): BDE-126 [5 bromine (Br)], BDE-177 (7 Br), BDE-195 (8 Br), and ^13^C_12_ BDE-209 (10 Br). Dust was extracted with 1:1 hexane:DCM, and extracts were analyzed using negative chemical ionization gas chromatography (GC)/mass spectrometry (MS) in multiple ion detection mode (see Supplemental Material, “Analytical method for quantification of the PBDEs”).

The method detection limit (MDL) was based on the concentration giving a peak approximately three times the matrix/instrument noise level. In < 1% of samples, PBDEs were quantified below the usual MDL; these were treated as detections in our analyses. The recoveries of the 4 SRSs were used to evaluate the method performance on a sample-by-sample basis. Recoveries from participants’ samples ranged from 66 ± 14% for SRS ^13^C_12_ BDE-209 to 78 ± 12% for BDE-126. In Supplemental Material, Table S1, we show mean sample recoveries for dust spikes and mean percent differences for duplicate samples. Mean low level spike recoveries ranged from 85% for BDE-208 to 122% for BDE-154. Mean percent differences in duplicates (*n* = 28) ranged from 4% for BDE-99 to 33% for BDE-207. The mean percent difference for BDE-207 included three pairs of samples with a detection in one sample and no detection in the duplicate; removing these gave a mean of 24%.

We used BDE-209 standards to estimate the rate of degradation of BDE-209 in the GC injector. Based on these percentages, we corrected the concentrations of BDE-206 through BDE-209 in participants’ samples. On a molar basis BDE-209 conversion was 3% to BDE-206, 2% to BDE-207, and 1% to BDE-208; we did not observe any conversion of BDE-209 to BDE-203. The correlations between concentrations corrected and uncorrected for BDE-209 degradation ranged from 0.63 for BDE-206 to 1.00 for BDE-209 (see Supplemental Material, Table S2). Analyses were based on the corrected concentrations.

*Statistical analysis*. Because PBDE concentrations had wide ranges and were right-skewed, natural logarithm transformations of concentrations were used in statistical analyses. For HVS3-sampled dust, we also calculated the chemical loading (nanograms per square meter), by multiplying the chemical concentration (nanograms per gram dust) by the dust loading [total fine dust collected divided by the sampled area (grams per square meter)]. Our results were similar when we used SRS-corrected and uncorrected concentrations; therefore, we report the uncorrected concentrations.

For samples with no detections of specific PBDEs, we used a single imputation method (Lubin et al. 2004) that selects values from the modeled (log-normal) distribution to assign values below the MDL. To create imputation models, we evaluated demographics, home characteristics, sampling year, and other factors in relation to PBDE concentrations by comparing medians and IQRs across levels of these variables. Factors associated with the PBDEs found at highest concentrations in homes (BDE-47, BDE-99, BDE-209) were sample year, time between diagnosis/reference date and dust sampling, residence location (urban, suburban, rural), income, and maternal age, but not season of dust sampling or sampling method. Imputation models included sample year and residence location—factors most strongly associated with PBDE concentrations.

In addition to evaluating the continuous PBDE concentration in relation to ALL risk, we categorized the distributions of most PBDEs into quartiles based on the distribution among controls. For BDE-196, BDE-203, BDE-207, and BDE-208 (detection rates < 80%) the lowest category was homes with no detections; concentrations above the MDL were categorized into tertiles. Odds ratios (OR) and 95% CIs were calculated using logistic regression. Tests for trend were based on the natural-log BDE concentration (or loading) in the regression models. The alpha level for statistical significance was 0.05.

We adjusted all analyses for age at diagnosis/reference date (< 1, 1 to < 2, 2 to 5, > 5 to < 8 years), sex, and race/ethnicity (non-Hispanic white, Hispanics, non-Hispanic other races), the original matching factors. We also adjusted for household income, year of dust sampling, and sampling method, factors that changed the OR for one or more PBDE by ≥ 10 percent. Adjustment for breastfeeding duration, child care attendance, mother’s or father’s education or age, age of carpets or residence, and geographic location (urban, suburban, rural) did not meet our criterion for confounding. We conducted stratified analyses to evaluate the consistency of associations by sex, child’s age at diagnosis/reference date, race/ethnicity, income, dust sampling method (HVS3 only), breastfeeding duration, and maternal age.

Although our analyses of BDE-206, BDE-207, BDE-208, and BDE-209 were based on the corrected concentrations, most studies of PBDEs and health have not corrected for BDE-209 degradation. Therefore, we show results for quartiles of the uncorrected concentrations of decaBDE in Supplemental Material, Table S3.

## Results

The MDLs, detection frequencies, and distributions of the PBDE concentrations among control homes are shown in Table 1. BDE-47 and BDE-99, the main congeners in the pentaBDE formulation, and BDE-209, the main constituent of the decaBDE formulation, were found at the highest concentrations in homes. The median concentration of BDE-183, the main congener in the octaBDE formulation, was > 50-fold lower than BDE-99 and BDE-209. Among controls and cases, the distributions of total PBDEs and individual PBDEs were similar by age, sex, race/ethnicity, and income level (not shown).

**Table 1:** Distribution of concentrations (ng/g)^*a*^ of PBDEs in tier 2 NCCLS dust samples among controls (*n* = 214).

PBDE congeners	No. of bromines	MDL (ng/g)	Percent detected	Median	IQR	Minimum	Maximum
PentaBDE
28	3	3.0	98	18	10–34	1.2	394
47	4	5.0	100	1,173	473–2,175	62.0	32,962
99	5	5.0	100	1,579	633–3,358	52.0	65,328
100	5	2.0	100	304	121–643	15.0	10,842
153	6	10.0	100	196	78–395	15.0	6,118
154	6	10.0	100	142	61–323	11.0	4,987
OctaBDE
183	7	2.0	100	17	11–29	1.1	987
196	8	2.0	63	5	1–11	0.1	84
197	8	2.0	85	8	4–14	0.2	494
203	8	3.0	71	10	3–19	0.1	304
DecaBDE^*b*^
206	9	20.0	90	28	15–41	0.2	443
207	9	0.5	74	7	0.4–20	0.02	396
208	9	0.5	76	4	0.4–10	0.004	156
209^*c*^	10	100.0	100	938	531–1,682	12.0	126,583
Sum of BDEs			100	5,269	2,965–8,926	304.0	131,325
^***a***^Percent detections were rounded to whole numbers; values below the MDL were imputed based on a log-normal distribution and accounting for residence location (urban, suburban, rural) and year of sampling. ^***b***^Concentrations of BDEs 206, 207, 208, and 209 were corrected for degradation in the GC/MS injector as described in “Methods”; percent detections for BDEs 206, 207, 208, and 209 were based on concentrations corrected for debromination of BDE-209 in the injector. ^***c***^Several samples had PBDEs quantified below the MDL.							

Congeners in the penta and octaBDE formulations were highly and significantly correlated within formulations [Spearman correlation coefficients (*r*_S_): pentaBDEs 0.79 to 0.997, octaBDEs 0.50 to 0.78] (see Supplemental Material, Table S4). PBDEs with 9 bromine atoms (BDE-206, BDE-207, BDE-208) were highly correlated (*r*_S_ = 0.79–0.81) but correlations with BDE-209 were lower (*r*_S_ = 0.13–0.16).

As shown in Table 2, most (62%) cases were male, with equal percentages (38%) being Hispanic and non-Hispanic white. Among controls, there were fewer Hispanics than non-Hispanic whites primarily because fewer Hispanic controls met the eligibility criterion of not moving since the reference date. A greater percentage of controls (50%) was in the highest income category (≥ $75,000) than cases (34%). Slightly more controls had dust sampled in the spring or summer (61%) than cases (53%). Cases and controls did not differ by residence age, location (urban, suburban, rural), sampling year, breastfeeding status, or maternal age at birth (not shown). The duration of residence at the sampled home before diagnosis/reference date [median (IQR)]: was 2.5 years (1.5–3.8) for cases and 2.4 years (1.6–4.1) for controls.

**Table 2:** Characteristics of ALL cases (*n* = 167) and controls (*n* = 214) included in analyses of PBDEs in carpet dust samples, 2001–2007 [*n* (%)].

Characteristic	Cases	Controls
Age (years)
< 1	2 (1)	5 (2)
1 to < 2	19 (11)	30 (14)
2–5	96 (57)	125 (58)
> 5	50 (30)	54 (25)
Sex
Male	104 (62)	125 (58)
Female	63 (38)	89 (42)
Ethnicity
Hispanic	63 (38)	63 (29)
Non-Hispanic White	64 (38)	103 (48)
Other races	40 (24)	48 (22)
Annual household income
$30,000–44,999	26 (16)	25 (12)
$45,000–59,999	30 (18)	28 (13)
$60,000–74,999	12 (7)	16 (7)
≥ $75,000	57 (34)	106 (50)
Season of sampling^*a*^
Fall or winter	78 (47)	83 (39)
Spring or summer	89 (53)	131 (61)
^***a***^Fall/winter included the months of October–March; spring/summer included April–September.		

Neither concentrations nor loadings of total PBDEs, nor the sum of PBDEs in each formulation, were associated with ALL risk (Table 3). None of the individual pentaBDE congeners was associated with risk of ALL. However, increasing residential concentrations of octaBDE congeners BDE-196 and BDE-203 were each associated with significantly increased risk of ALL (BDE-196: OR = 1.20, 95% CI: 1.01, 1.42; BDE-203: OR = 1.25, 95% CI: 1.05, 1.48). Results were similar for PBDE loadings (HVS3 samples: 101 cases, 133 controls). ORs were elevated for a 1-ln(ng/g) change in concentration and loading of BDE-206, BDE-207, and BDE-208 but did not reach statistical significance. There was no association with BDE-209.

**Table 3:** ORs (95% CIs) for acute lymphoblastic leukemia associated with the natural log of PBDE concentrations and loadings.^*a*^

PBDE	Concentration (ng/g)^*b*^	Loadings (ng/m^2^)^*b*^
Total PBDEs	0.82 (0.66, 1.03)	0.84 (0.68, 1.05)
Sum of pentaBDEs	0.86 (0.71, 1.05)	0.88 (0.72, 1.08)
28	0.87 (0.70, 1.09)	0.92 (0.74, 1.14)
47	0.87 (0.71, 1.05)	0.88 (0.72, 1.08)
99	0.86 (0.71, 1.03)	0.87 (0.72, 1.06)
100	0.85 (0.70, 1.03)	0.88 (0.72, 1.07)
153	0.88 (0.72, 1.07)	0.90 (0.74, 1.10)
154	0.84 (0.69, 1.02)	0.86 (0.71, 1.06)
Sum of octaBDEs	1.08 (0.85, 1.37)	1.02 (0.82, 1.28)
183	0.94 (0.75, 1.18)	0.95 (0.76, 1.18)
196	1.20 (1.01, 1.42)	1.19 (1.00, 1.41)
197	1.10 (0.91, 1.33)	1.04 (0.85, 1.28)
203	1.25 (1.05, 1.48)	1.17 (0.98, 1.40)
Sum of DecaBDEs	0.95 (0.78, 1.17)	0.93 (0.77, 1.14)
206	1.16 (0.91, 1.48)	1.10 (0.88, 1.38)
207	1.10 (0.99, 1.22)	1.09 (0.96, 1.24)
208	1.11 (0.99, 1.25)	1.08 (0.93, 1.26)
209	0.95 (0.78, 1.15)	0.93 (0.77, 1.13)
^***a***^Loading analysis based on 101 ALL cases and 133 controls with HVS3 dust samples. ^***b***^OR for a 1-unit change in the natural logarithm of the concentration or loading; adjusted for child’s age, sex, and race/ethnicity, family income, sample year, sampling method (except loadings analyses, which were based on HVS3 dust only).		

Results for categorical analyses of PBDE concentrations are shown in Table 4. We observed elevated ORs for the second quartiles of the summed pentaBDEs and for BDE-47, BDE-99, BDE-100, BDE-153, and BDE-154 but little or no increase for the third or fourth quartiles. Results were similar for pentaBDE loadings (not shown). Risk was not significantly elevated for the summed octaBDEs and decaBDEs but was significantly increased for specific congeners in these formulations. Compared with no detections, risk increased with increasing tertiles of BDE-196, BDE-203, BDE-207, and BDE-208 and was significantly increased in the highest tertiles for BDE-196 (OR = 2.08; 95% CI: 1.14, 3.77), BDE-203 (OR = 1.98; 95% CI: 1.09, 3.62), BDE-206 (OR = 2.08; 95% CI: 1.08, 3.98), and BDE-207 (OR = 1.97; 95% CI: 1.03, 3.76). We observed similar associations and positive trends for PBDE loadings (not shown).

**Table 4:** ORs (95% CIs) for the association of quartiles or tertiles of PBDE concentrations in the penta, octa, and deca formulations and risk of ALL.

PBDE congener (ng/g)	Controls	Cases	OR (95% CI)^*a*^	*p*-Trend^*b*^
Sum of pentaBDEs
< 1,475	54	38	1.0
1,475 to < 3,325	53	52	1.49 (0.83, 2.67)
3,325–7,261	54	46	1.21 (0.67, 2.18)
> 7,261	53	31	0.70 (0.37, 1.32)	0.133
BDE-28
ND < 10	57	53	1.0
10 to < 18	52	41	0.76 (0.43, 1.37)
18–34	53	37	0.75 (0.42, 1.35)
> 34	52	36	0.69 (0.38, 1.24)	0.217
BDE-47
< 473	54	37	1.0
473 to < 1,173	53	58	1.64 (0.92, 2.93)
1,173–2,175	54	35	0.94 (0.51, 1.74)
> 2,175	53	37	0.92 (0.50, 1.70)	0.148
BDE-99
< 633	54	36	1.0
633 to < 1,579	53	54	1.60 (0.89, 2.88)
1,579–3,358	54	46	1.26 (0.70, 2.29)
> 3,358	53	31	0.72 (0.38, 1.37)	0.103
BDE-100
< 121	54	37	1.0
121 to < 304	53	56	1.60 (0.89, 2.88)
304–643	54	40	1.06 (0.58, 1.94)
> 643	53	34	0.79 (0.42, 1.48)	0.099
BDE-153
ND to < 78	54	34	1.0
78 to < 196	53	57	1.82 (1.01, 3.28)
196–395	54	42	1.20 (0.65, 2.20)
> 395	53	34	0.89 (0.47, 1.67)	0.187
BDE-154
ND–61	54	38	1.0
61 to < 142	53	53	1.45 (0.80, 2.60)
142–323	54	46	1.19 (0.66, 2.15)
> 323	53	30	0.64 (0.34, 1.22)	0.076
Sum of octaBDEs
< 17.2	54	40	1.0
17.2 to < 43.4	53	38	1.00 (0.55, 1.85)
43.4–55.6	54	45	1.16 (0.65, 2.08)
> 55.6	53	44	1.27 (0.70, 2.30)	0.518
BDE-183
< 11	55	53	1.0
11 to < 17	53	41	0.71 (0.40, 1.26)
17–29	53	27	0.55 (0.30, 1.01)
> 29	53	46	0.92 (0.53, 1.61)	0.618
BDE-196
ND	79	45	1.0
> 1 to < 6.3	45	38	1.30 (0.72, 2.33)
6.3–11.4	45	39	1.52 (0.85, 2.71)
> 11.4	45	45	2.08 (1.14, 3.77)	0.034
BDE-197
ND to < 4.5	54	44	1.0
4.5 to < 8.2	53	38	0.89 (0.49, 1.61)
8.2–14.1	54	41	1.01 (0.56, 1.81)
> 14.1	53	44	1.20 (0.67, 2.18)	0.319
BDE-203
ND	62	34	1.0
> 1 to < 11	51	39	1.35 (0.73, 2.48)
11–20	50	46	1.79 (0.98, 3.28)
> 20	51	48	1.98 (1.09, 3.62)	0.011
Sum of decaBDEs
< 569	54	45	1.0
569 to < 989	53	51	1.34 (0.76, 2.38)
989–1,789	54	33	0.84 (0.46, 1.54)
> 1,789	53	38	1.01 (0.56, 1.84)	0.630
BDE-206
ND	54	31	1.0
15 to < 28	53	47	1.73 (0.94, 3.19)
28–41	54	39	1.67 (0.88, 3.19)
> 41	53	50	2.08 (1.08, 3.98)	0.224
BDE-207
ND	56	34	1.0
1 to < 7.8	53	41	1.23 (0.67, 2.26)
7.8–19.7	52	47	1.96 (1.05, 3.67)
> 19.7	53	45	1.97 (1.03, 3.76)	0.065
BDE-208
ND	52	33	1.0
0.5 to < 4.3	54	44	1.27 (0.69, 2.36)
4.3–10.0	53	39	1.41 (0.75, 2.66)
> 10.0	55	51	1.75 (0.94, 3.24)	0.082
BDE-209
< 534	55	48	1.0
534 to < 947	53	49	1.23 (0.69, 2.17)
947–1,682	53	33	0.80 (0.44, 1.45)
> 1,682	53	37	0.93 (0.51, 1.68)	0.573
ND, not detected. ^***a***^ORs adjusted for age, sex, race/ethnicity, family income, sampling year, sampling method (HVS3 or vacuum bag). ^***b***^*p*-Value for trend based on continuous form of the variable (ng/g).				

Risk estimates for PBDEs were not changed by adjustment for residential concentrations of PCBs (not shown), which were associated with increased ALL risk in this population (Ward et al. 2009). Our results were similar when we restricted our analyses to those who lived at their home for at least 1 year before diagnosis/reference date, to subjects whose homes were sampled within 2 years of diagnosis/reference date, or to HVS3 dust (not shown). We observed consistent results by age, sex, race/ethnicity, maternal age, and breastfeeding status (not shown).

Our primary analyses of decaBDEs were based on concentrations corrected for BDE-209 degradation. Correction resulted in a small increase (5%) in the median BDE-209 concentration. Compared with uncorrected medians, corrected concentrations of BDE-206, BDE-207, and BDE-208 decreased about 49%, 75%, and 70%, respectively. Rank correlations between corrected and uncorrected concentrations were 0.63, 0.66, and 0.69 for BDE-206, BDE-207, and BDE-208, respectively; whereas corrected and uncorrected BDE-209 concentrations had a correlation of 1.00 (see Supplemental Material, Table S2). The associations we observed between the corrected BDE-207 and BDE-208 concentrations and ALL risk were monotonic and demonstrated stronger positive trends than the associations based on the uncorrected concentrations (tertiles above the detection limit vs. nondetections; see Supplemental Material, Table S3).

## Discussion

We found no association with ALL risk for the total summed PBDEs, for the sum of congeners in the penta, octa, and decaBDE formulations, or for the dominant constituents of these formulations (BDEs 99, 183, and 209, respectively). However, children whose homes had the highest concentrations of specific, less common PBDEs with 8 or 9 bromine atoms (BDE-196, BDE-203, BDE-206, BDE-207) had about twice the risk of ALL compared with children in homes with low or nondetected levels of these PBDEs. Results were similar, although not all associations were statistically significant, for PBDE loadings in the subgroup with HVS3 dust. Adjustment for BDE-209 degradation in the GC injector resulted in stronger positive trends for BDE-206, BDE-207, and BDE-208 compared with analyses of unadjusted concentrations of these congeners, suggesting that adjustment may have improved exposure classification.

To our knowledge, no prior epidemiologic studies have evaluated residential PBDE exposure and childhood cancer risk. In animals, only the carcinogenicity of BDE-209 has been evaluated; increases in thyroid gland follicular cell adenomas and carcinomas (combined) were observed for male and female mice, and dose-related increases in liver adenomas were found in male and female rats [National Toxicology Program (NTP) 1986]. These findings were described as equivocal by the NTP (1986); however, the U.S. EPA considered them to be suggestive of carcinogenic potential (U.S. EPA 2008). Chronic long-term carcinogenicity studies of the pentaBDEs are currently being conducted by the NTP (2014).

BDE-209 is less easily absorbed and more rapidly excreted than lower-brominated PBDEs (Birnbaum and Cohen Hubal 2006); therefore, toxicities may be different from other PBDEs (Birnbaum and Cohen Hubal 2006; Dunnick and Nyska 2009). It is likely that PBDE congeners differ in toxicities and carcinogenic potential because differing toxicities are well-documented for the structurally similar PCBs, furans, and dioxins (Lorber 2008). However, to date, there is limited information on the toxicities of the octa and nonaBDEs (Birnbaum and Cohen Hubal 2006; Lorber 2008). Most short-term toxicity studies have focused on pentaBDE formulation congeners, and have demonstrated toxic effects on the liver and thyroid gland (Dunnick and Nyska 2009; Dunnick et al. 2012). Lower-brominated PBDEs also have endocrine-disrupting properties similar to dioxins (Fowles et al. 1994). PBDEs can be contaminated with dioxins and furans (Ren et al. 2011); however, tetrachlorodibenzo-*p*-dioxin (TCDD) at contaminant levels has not shown toxicity to the liver and thyroid gland to the same extent as the PBDEs (Suzuki et al. 2010). Nevertheless, the dioxin-like activity of the PBDEs is a concern and a possible mechanism for carcinogenicity. Further mechanistic studies of specific PBDE congeners would be informative.

Studies of the immunotoxicity of PBDEs are quite limited. A mouse study showed adverse effects of BDE-99 and Bromkal 70-5 DE, a commercial pentaBDE mixture, on cytokine and chemokine levels (Lundgren et al. 2009). A recent animal study (Fair et al. 2012) of DE-71, a commercial pentaBDE mixture, found increased lymphocyte proliferation, decreased NK cell activity, and decreased numbers of peripheral monocytes. A cross-sectional study of Dutch adolescents found decreased lymphocytes associated with higher serum concentrations of summed congeners in the penta and octaBDE formulations (Leijs et al. 2009). The clinical implications of these effects are unknown. Chemicals that perturb the immune system may increase chances of an inappropriate response to infection, which is a hypothesized cause of childhood leukemia (Greaves 1988).

Blood levels of PBDEs in California children (2003–2005) (Rose et al. 2010), first-time California mothers (similar time frame) (Park et al. 2011), and Mexican-American children in California (2007–2008) (Bradman et al. 2012) are among the highest in the world. Levels of BDE-47 in breast milk (Park et al. 2011) exceeded the U.S. EPA reference dose for neurodevelopmental and liver toxicity for 60% and 7% of mothers, respectively. Given the potential for high exposure to the developing child, further research on the health effects of PBDEs is warranted.

PCBs, which are structurally similar to PBDEs, are immunotoxic (Heilmann et al. 2010) and considered to be probable human carcinogens by the International Agency for Research on Cancer (Lauby-Secretan et al. 2013) and the U.S. EPA (1996). We observed a positive association between residential levels of PCBs and ALL risk in this study population (Ward et al. 2009); however, concentrations of PBDEs and PCBs were not significantly correlated in our study, and adjustment for PCBs levels did not change the associations with PBDE congeners. Similarly, studies of Hispanic women with young children in Salinas County, California (Bradman et al. 2007), and first-time mothers in California (Park et al. 2011), found no significant correlation between concentrations of PCBs and PBDEs in maternal blood or breast milk, suggesting different routes and sources of exposure. Differences in the time periods of peak production and differences in uses may account for the low correlations between PCB and PBDEs in homes (Harrad et al. 2008).

Strengths of our study were rapid case ascertainment, population-based selection of controls, and high participation rates. An additional strength was the measurement of numerous chemicals that allowed us to adjust for co-occurrence with other chemicals in the homes. We were able to evaluate PBDE loadings among cases and controls with HVS3 dust, and we observed results that were similar to PBDE concentrations. Loadings are postulated to be a more accurate indicator of exposure for small children (Bradman et al. 1997; Lanphear et al. 1998), for whom dust ingestion is a major route of exposure. A previous study (Allen et al. 2008) found low correlations between PBDE concentrations in household vacuum bags and interviewer-collected dust. In our study, concentration ranges and associations with ALL were similar for HVS3- and vacuum-collected dust samples combined and for HVS3-collected dust samples only, providing some evidence that concentrations of PBDEs in household vacuum dust may be a useful exposure indicator.

A limitation of our study was that we measured PBDEs in carpet dust only. However, a few studies have measured PBDEs in both dust and serum and found dust to be an important source of exposure for first-time mothers and young children (Rose et al. 2010; Stapleton et al. 2012; Wu et al. 2007). Further, a recent study in New Zealand (Coakley et al. 2013) found significant correlations between PBDE concentrations in dust from mattresses and floors and mothers’ breast milk. Another limitation was our use of one dust sample to characterize exposure, which may result in exposure misclassification if PBDE levels vary over time. In a subset of homes in our study (Whitehead et al. 2013), two dust samples from household vacuums taken 3–8 years apart and analyzed for PBDEs by a different laboratory in 2010–2012 had moderate Spearman rank correlations (range, 0.16–0.56). Correlations were generally higher for samples 3–6 years apart compared with 7–8 years and were higher for the lower-brominated PBDEs compared with the higher-brominated PBDEs (e.g., pentaBDE *r*_S_ > octaBDE *r*_S_ > decaBDE *r*_S_). A prior study (Allen et al. 2008) demonstrated strong correlations between PBDEs measured in two dust samples taken about 8 months apart. Both studies provide some support for the usefulness of one dust sample to characterize residential exposure over a period of a few years. Further limitations included the small sample size, especially for analyses of HVS3 dust, and relatively small numbers of Hispanics and lower-income participants in our sampled population. However, we found little evidence that PBDE levels varied substantially by race/ethnicity or income levels. Results among Hispanics and non-Hispanic whites and stratified by income level were similar to results overall, providing some assurance that our findings were not biased by these factors.

In summary, we found no association with ALL for most common PBDEs in residential dust, but observed positive associations for specific octa and nonaBDEs. Among those with residential concentrations in the highest tertile of specific octaBDE congeners (BDE-196 and BDE-203) and decaBDE congeners (BDE-206 and BDE-207), we observed a significantly increased risk of ALL. Positive trends for BDE-206, BDE-207, and BDE-208 were stronger after adjusting for BDE-209 degradation in the GC injector. Additional animal carcinogenicity and toxicity studies and epidemiologic studies with repeated sampling and biological measures of exposure would be informative.

## Supplemental Material

(386 KB) PDF
