# Supplementary material for: Residential Levels of Polybrominated Diphenyl Ethers and Risk of Childhood Acute Lymphoblastic Leukemia in California
Source: Environ Health Perspect. 2014 Jun 3;122(10):1110–6. doi: 10.1289/ehp.1307602 (PMC4181922; doi:10.1289/ehp.1307602)
Supplement: (386 KB) PDF [file ehp.1307602.s001.508.pdf]

Supplemental Material

Residential Levels of Polybrominated Diphenyl Ethers and Risk of Childhood Acute Lymphoblastic Leukemia in California

Mary H. Ward, Joanne S. Colt, Nicole C. Deziel, Todd P. Whitehead, Peggy Reynolds, Robert B. Gunier, Marcia Nishioka, Gary V. Dahl, Stephen M. Rappaport, Patricia A. Buffler, and Catherine Metayer

Table of Contents

Analytical method for quantification of the PBDEs - Page 2

Supplemental Material, Table S1: Method Detection Limits (MDL) and quality control results for solvent blanks, percent recovery of BDE-spiked dust, and relative percent difference for duplicate dust samples - Page 4

Supplemental Material, Table S2: Spearman correlations between corrected and uncorrected concentrations (ng/g) of BDE 206-209 in dust samples, controls (n=214) - Page 5

Supplemental Material, Table S3: Odds Ratios (ORs) and 95% Confidence Intervals (CI) for the association of uncorrected DecaBDE concentrations and risk of ALL - Page 6

Supplemental Material, Table S4: Spearman correlations between concentrations (ng/g) of 14 PBDEs in carpet dust samples, controls (n=214) - Page 7

Analytical method for quantification of the PBDEs

Each dust sample was sonified in 12 mL of 1:1 hexane:DCM twice, with the majority of the extract drawn off after each extraction. The extract was treated with 1 g of acid silica for 1 hr on a shaker table. After decanting the treated extract, the acid silica was rinsed 3 additional times with the extraction solvent. The extract was concentrated to 1 mL, solvent exchanged into hexane, and spiked with the internal standard 13C12 BDE-138.

Extracts were analyzed using negative chemical ionization GC/MS in the multiple ion detection mode, with methane as the reagent gas (Agilent 5973N MS with HP6890 GC). The GC conditions included a 15 m DB-5ms column (0.10µm film thickness, 0.25 mm diameter) programmed from 100-200 C at 10 C/min and then 200-300 C at 30 C/min; the injector temperature was 290 C. Sample extracts were analyzed concurrently with a 6-point calibration curve that spanned the range of 2-500 ng/mL for most PBDEs and 4-1000 ng/mL for BDEs 203 through 209. Analytes were quantified using the internal standard method against linear least squares regression calibration curves. For samples with a concentration more than 15% over the maximum calibration curve point, the sample was diluted and re-spiked proportionally with the internal standard, and reanalyzed. Concentrations were reported as ng/g both with and without Surrogate Recovery Standards (SRS) correction (see Supplemental Material, Table S1, footnote c for SRS matches).

Preliminary analyses showed that the extent of thermal degradation of BDE-209 in the GC injector, forming BDE-206, BDE-207 and BDE-208, was the same whether an injector temperature of 260 or 290 C was used; however, at 260 C, the mass transfer from injector to column was about one-tenth of that observed at 290 C. Thus, we used an injector temperature of 290 C. We determined the percentage of BDE-209 that was converted to specific octa and

nonaBDEs in the GC injector by spiking three BDE-209 concentrations (100, 500, 1000 ng) into solvent and computing the average for each BDE congener. Concentrations of 4 and 20 ng/mL were also used but no degradation to BDE-206, BDE-207, or BDE-208 was detected. Conversion rates (mean $\pm$ standard deviation) were: 2.8 $\pm$ 1.2% for BDE-206, 1.7 $\pm$ 0.3% for BDE-207, and 0.95 $\pm$ 0.1% for BDE-208. We corrected for the conversion of BDE-209 in each sample as follows: on a molar basis, 3% to BDE-206, 2% to BDE-207, and 1% to BDE-208; we did not observe any conversion of BDE-209 to BDE-203.

The quality control results for solvent method blanks, low and high level analyte spikes to dust, and duplicate samples are shown in Supplemental Material, Table S1. The method detection limit (MDL) was based on the concentration giving approximately 3 times the matrix/instrument noise level (which were similar because of the high degree of sample cleanup). We estimated that there was about 0.5% unlabeled BDE-209 in the labeled SRS (13C12 BDE 209). Because of this low contribution to BDE 209 and the high native levels of BDE 209, we did not correct for this in the actual samples. The recoveries of the 4 SRSs served as important metrics to evaluate method performance on a sample-by-sample basis and to correct the concentrations of the matched PBDEs (i.e., those possessing similar numbers of bromines; see Table 1, footnote c). Recoveries of SRS BDE-126 in samples (n=448), spikes (n=28), and duplicates (n=56) were: 78 ± 12%, 78 ± 11%, and 80 ± 12%, respectively. Recoveries of SRS BDE-177 in samples, spikes, and duplicates were: 69 ± 10%, 73 ± 12%, and 70 ± 11%, respectively. Recoveries of SRS BDE-195 in samples, spikes, and duplicates were: 68 ± 12%, 71 ± 11%, and 69 ± 11%, respectively. Recoveries of SRS $^{13}C_{12}$ BDE-209 in samples, spikes, and duplicates were: 66 ± 14%, 70 ± 15%, and 66 ± 14%, respectively.

Table S1. Method Detection Limits (MDL) and quality control results for solvent blanks, percent recovery of BDE-spiked dust, and relative percent difference for duplicate dust samples.

Analyte	MDL in dust, ng/g	Solvent method blank ng/g, mean ± SD (n=28)	% Recovery for low level dust spike, mean ± SD (n=14) (native analyte concentration range in spiked samples, ng/g)[a,b,c]	% Recovery for high level dust spike, mean ± SD (n=14) (native analyte concentration range in spiked samples, ng/g)[c,d]	Relative percent difference for duplicates, mean (n= 28)
BDE 28	3	0.3 ± 1.6	104 ± 12 (4-200)	111 ± 11 (5-100)	10%
BDE 47	5	2.1 ± 4.5	95 ± 36 (350-800)	111 ± 12 (175-3800)	5%
BDE 99	5	3.3 ± 7.9	92 ± 24 (240-1100)	112 ± 12 (250 (6800)	4%
BDE 100	2	0.4 ± 1.2	103 ± 16 (55-200)	111 ± 11 (50-1100)	5%
BDE 153	10	ND[e]	118 ± 35 (20-1750)	115 ± 13 (25-1200)	7%
BDE 154	10	ND	122 ± 31 (20-1400)	120 ± 14 (20-500)	5%
BDE 183	2	0.5 ± 1.5	110 ± 17 (5-80)	110 ± 10 (5-60)	13%
BDE 196	2	1.5 ± 4.4	100 ± 9 (ND-15)	98 ± 15 (ND-30)	17%
BDE 197	2	1.8 ± 4.7	99 ± 20 (5-25)	96 ± 13 (ND-40)	23%
BDE 203	3	2.7 ± 7.0	90 ± 11 (ND-25)	96 ± 17 (ND-35)	21%
BDE 206	20	27.5 ± 20.1	88 ± 29 (ND-70)	86 ± 23 (ND-100)	11%
BDE 207	0.5	3.5 ± 6.2	93 ± 37 (ND-35)	84 ± 22 (ND-70)	33%[f]
BDE 208	0.5	5.0 ± 6.1	85 ± 25 (ND-25)	82 ± 16 (ND-50)	23%
BDE 209	100	ND	88 ± 74 (120-1400)	83 ± 27 (250-2700)	11%

SD=standard deviation.

[a]Fortification level of 250 ng/g for BDEs 28, 47, 99, 100, 153, 154, 183; fortification level of 350 ng/g for BDEs 196, 197; fortification level of 500 ng/g for BDEs 203, 206, 207, 208, 209. [b]n=6 for BDEs 47, 99, 100, and 209; native levels too high in several samples to ascertain low spike level recovery. [c]Recoveries reported with SRS correction; recovery of SRS BDE 126 used to correct recovery of BDEs 28, 47, 99, 100; recovery of SRS BDE 177 used to correct recovery of BDEs 153, 154, 183; recovery of SRS BDE 195 used to correct recovery of BDEs 196, 197, 203; recovery of SRS BDE $^{13}C_{12}$ BDE 209 used to correct recovery of BDEs 206, 207, 208, 209. [d]Fortification level of 1250 ng/g for BDEs 28, 47, 99, 100, 153, 154, 183; fortification level of 1750 ng/g for BDEs 196, 197; fortification level of 2500 ng/g for BDEs 203, 206, 207, 208, 209. [e]ND= not detected, all less than MDL. [f]Includes three pairs with a detection in one sample and no detection in the duplicate. Excluding these three pairs, the mean relative percent difference was 24%.

Table S2. Spearman correlations between corrected[a] and uncorrected concentrations (ng/g) of BDE 206-209 in dust samples, controls (n=214).

PBDE congener	Spearman correlation coefficients	p-value
BDE-206	0.63	<0.0001
BDE-207	0.66	<0.0001
BDE-208	0.69	<0.0001
BDE-209	1.00	<0.0001

[a]Corrected for estimated degradation of BDE-209 in the GC injector; values below the detection limited were imputed assuming a lognormal distribution

Table S3. Odds Ratios (ORs) and 95% Confidence Intervals (CI) for the association of uncorrected DecaBDE concentrations and risk of ALL.

BDE (ng/g)	Controls	Cases	OR (95% CI)[a]	P-trend[b]
**Sum of DecaBDEs**				
<569	54	45	1.0	
569-<987	53	51	1.35 (0.76, 2.38)	
987-<1762	54	33	0.84 (0.45, 1.54)	
1762+	53	38	1.01 (0.56, 1.84)	0.645
**BDE206**				
ND-37	54	35	1.0	
37-<55	53	39	1.52 (0.81, 2.86)	
55-86	54	52	2.09 (1.13, 3.86)	
>86	53	41	1.56 (0.83, 2.95)	0.668
**BDE207**				
ND-<14.5	54	34	1.0	
14.5-<27.7	53	49	2.02 (1.09, 3.77)	
27.7-48.2	54	45	1.84 (0.97, 3.47)	
>48.2	53	39	1.75 (0.90, 3.37)	0.365
**BDE208**				
ND-<7.2	54	29	1.0	
7.2-<13.5	53	40	1.89 (0.98, 3.62)	
13.5-27.5	54	63	3.07 (1.64, 5.75)	
>27.5	53	35	1.74 (0.89, 3.40)	0.113
**BDE209**				
<503	55	48	1.0	
503-<893	53	49	1.23 (0.69, 2.17)	
893-1587	53	33	0.80 (0.44, 1.45)	
>1587	53	37	0.93 (0.51, 1.68)	0.573

[a]ORs adjusted for age, sex, race/ethnicity, family income, sampling year, dust collection method (HVS3 or vacuum bag). [b]P-value for trend based on continuous form of the variable (ng/g).

Table S4. Spearman correlations between concentrations (ng/g)[a] of 14 BDEs in carpet dust samples, controls (n=214).

	BDE-47	BDE-99	BDE-100	BDE-153	BDE-154	BDE-183	BDE-196	BDE-197	BDE-203	BDE-206	BDE-207	BDE-208	BDE-209
**BDE-28**	0.87<br><.0001	0.81<br><.0001	0.82<br><.0001	0.79<br><.0001	0.79<br><.0001	0.49<br><.0001	0.10<br>0.1365	0.20<br>0.004	0.10<br>0.150	0.19<br>0.006	0.17<br>0.014	0.18<br>0.010	0.07<br>0.341
**BDE-47**	1.0	0.98<br><.0001	0.98<br><.0001	0.93<br><.0001	0.94<br><.0001	0.51<br><.0001	0.07<br>0.337	0.15<br>0.033	0.04<br>0.518	0.10<br>0.129	0.11<br>0.125	0.09<br>0.203	-0.01<br>0.887
**BDE-99**		1.0	0.997<br><.0001	0.97<br><.0001	0.98<br><.0001	0.49<br><.0001	0.05<br>0.490	0.11<br>0.119	0.02<br>0.751	0.08<br>0.240	0.07<br>0.309	0.06<br>0.416	-0.02<br>0.721
**BDE-100**			1.0	0.96<br><.0001	0.98<br><.0001	0.50<br><.0001	0.05<br>0.473	0.12<br>0.089	0.03<br>0.711	0.09<br>0.212	0.08<br>0.259	0.06<br>0.373	-0.03<br>0.638
**BDE-153**				1.0	0.98<br><.0001	0.53<br><.0001	0.07<br>0.296	0.12<br>0.080	0.04<br>0.561	0.08<br>0.269	0.08<br>0.273	0.05<br>0.509	-0.03<br>0.651
**BDE-154**					1.0	0.50<br><.0001	0.04<br>0.567	0.09<br>0.180	0.009<br>0.900	0.04<br>0.545	0.03<br>0.653	0.02<br>0.824	-0.02<br>0.792
**BDE-183**						1.0	0.50<br><.0001	0.70<br><.0001	0.55<br><.0001	0.20<br>0.004	0.38<br><.0001	0.20<br>0.004	0.16<br>0.016
**BDE-196**							1.00	0.68<br><.0001	0.78<br><.0001	0.39<br><.0001	0.53<br><.0001	0.40<br><.0001	0.23<br>0.0006
**BDE-197**								1.0	0.73<br><.0001	0.38<br><.0001	0.56<br><.0001	0.41<br><.0001	0.33<br><.0001
**BDE-203**									1.00	0.45<br><.0001	0.60<br><.0001	0.44<br><.0001	0.32<br><.0001
**BDE-206**										1.0	0.79<br><.0001	0.81<br><.0001	0.16<br>0.020
**BDE-207**											1.0	0.79<br><.0001	0.13<br>0.052
**BDE-208**												1.0	0.14<br>0.043

BDE=polybrominated diphenyl ethers

Penta-BDEs: BDE-28, BDE-47, BDE-99, BDE-100, BDE-153, BDE-154

Octa-BDEs: BDE-183, BDE-196, BDE-197, BDE-203

Deca-BDEs: BDE-206, BDE-207, BDE-208

[a]Values below the MDL were imputed based on a lognormal distribution and accounting for residence location (urban, suburban, rural) and year of sampling.
